# Network Analysis of Rat Spatial Cognition: Behaviorally-Established Symmetry in a Physically Asymmetrical Environment

**DOI:** 10.1371/journal.pone.0040760

**Published:** 2012-07-18

**Authors:** Shahaf Weiss, Osnat Yaski, David Eilam, Juval Portugali, Efrat Blumenfeld-Lieberthal

**Affiliations:** 1 Department of Zoology, George Wise Faculty of Life Sciences, Tel Aviv University, Tel-Aviv, Israel; 2 Department of Geography and the Human Environment, The Lester and Sally Entin, Faculty of the Humanities, Tel Aviv University, Tel-Aviv, Israel; 3 The David Azrieli School of Architecture, Yolanda and David Katz Faculty of the Arts, Tel Aviv University, Tel-Aviv, Israel; 4 Department of OTANES, University of South Africa, Pretoria, South Africa; Utrecht University, The Netherlands

## Abstract

**Background:**

We set out to solve two inherent problems in the study of animal spatial cognition (i) What is a “place”?; and (ii) whether behaviors that are not revealed as differing by one methodology could be revealed as different when analyzed using a different approach.

**Methodology:**

We applied network analysis to scrutinize spatial behavior of rats tested in either a symmetrical or asymmetrical layout of 4, 8, or 12 objects placed along the perimeter of a round arena. We considered locations as the units of the network (nodes), and passes between locations as the links within the network.

**Principal Findings:**

While there were only minor activity differences between rats tested in the symmetrical or asymmetrical object layouts, network analysis revealed substantial differences. Viewing ‘location’ as a cluster of stopping coordinates, the key locations (large clusters of stopping coordinates) were at the objects in both layouts with 4 objects. However, in the asymmetrical layout with 4 objects, additional key locations were spaced by the rats between the objects, forming symmetry among the key locations. It was as if the rats had behaviorally imposed symmetry on the physically asymmetrical environment. Based on a previous finding that wayfinding is easier in symmetrical environments, we suggest that when the physical attributes of the environment were not symmetrical, the rats established a symmetric layout of key locations, thereby acquiring a more legible environment despite its complex physical structure.

**Conclusions and Significance:**

The present study adds a behavioral definition for “location”, a term that so far has been mostly discussed according to its physical attributes or neurobiological correlates (e.g. - place and grid neurons). Moreover, network analysis enabled the assessment of the importance of a location, even when that location did not display any distinctive physical properties.

## Introduction

The ability of animals to become organized in time and space rests, at least partially, on their perception of direction and distance between landmarks and self [1], [2], [3]. Landmarks constitute external cues in the layout of the environment, in reference to which the animal is able to locate itself [4], [5], [6], [7]. Animals may also use internal cues generated by their self-movement (e.g. vestibular and kinesthetic cues), with the navigator continuously integrating and updating its position in reference to a fixed location; for example, the starting point of travel [8], [9], [10], [11]. Both external and internal cues are utilized by animals in navigating and constructing spatial representation [8], [12], [13], [14], [15]. Once the representation of the environment has been acquired, animals can switch to locale navigation [2]; that is, to identifying origin, destination, directions, and distances [16]. The spatial representation may be viewed as a set of connected places (e.g. landmarks, food sites, home, den, etc.) that are systematically related to each other [2]. The advantage of using locale navigation lies in the flexibility and ability to navigate from anyplace to anyplace within the represented space, while allowing the selection of many possible routes leading to the goal. In the context of this general theme of animal spatial cognition, the present study aimed at solving two inherent problems.

### Problem I: What is a “Place”?

The keystones of spatial representation and map navigation are the different places (locales) in the environment. However, the study of spatial behavior typically refers to places as mere physical entities, defining them according to their physical properties. In nature, places are typically meaningful and physically distinctive (den, food patch, water source), whereas in experimental environments they are usually ambiguous (except for the targets in goal-directed navigation tasks, such as a maze). Indeed, the common approach in laboratory environments is to predefine physical places such as perimeter, center, corners, objects, or zones of a superimposed grid [17], [18], [19], [20], [21]. For example, a grid division imposed on an open-field apparatus by lines or photobeams usually serves in measuring the time spent in each zone of the grid, the number of visits paid to each zone, or lines crossed during travel [22], [23], [24], [25], [26]. Altogether, behavior in experimental settings is usually measured in relation to a set of predefined places. The problem arises, however, when a mismatch occurs between behavior and the predefined locations. For example, when an animal stops frequently on the border between two adjacent predefined zones, the “behavioral location” could be small, but it spreads over the two zones. Another example is that of the zones along arena walls [22] or around objects [21], where the impact of the walls/objects extends further away from the predefined zones of these landmarks [27], [28]. Indeed, it is the animal’s behavior that confers identity and meaning upon a landmark or other physical properties of the environment, as noted by Wise [29]: *“It (space) is marked physically, with objects forming borders, walls and fences… The marker (wall, road, line border, post, sign) is static, dull and cold. But when lived (encountered, manipulated, touched, voiced, glanced at, practised), it radiates a milieu, a field of force, a shape of space”*. Accordingly, the first aim of the present study was to seek a behavioral definition for locations in the environment. In other words, we sought to delineate locations in the environment according to their manifestation in the spatial behavior of the rats, and not only according to their physical properties.

### Problem II: Understanding Spatial behavior has been Limited by the Available Analytic Tools

One approach in studying spatial behavior is to train the animals to reach a goal with reference to specific cues, and then to alter these cues [30], [31], [32]. Another approach is to track a freely-moving animal in an unfamiliar environment, and from its movements and routes to draw conclusions regarding the representation of the environment [33], [34], [35], [36], [37], [38]. The latter approach, also utilized in the present study, is confined by its analytic means. Specifically, manual scoring or video tracking can provide the traveled distance, locomoting time, the time spent in a specific sector of the environment, etc [17], [18], [19]. Nevertheless, behaviors that are not revealed as differing when analyzed by the common means (manual scoring, video tracking), could reveal differences when analyzed using a different approach. In the present study, we applied video-tracking together with topologic network analysis, in the search for previously undetected characteristics of spatial behavior in rats.

Network analysis is concerned with topologic mapping of the relations among interconnected units (the network nodes). The network can represent social interactions [39], biological links (e.g. neural network [40]), or man-made systems (e.g. infrastructure [41]). Network analysis typically focuses on the interactions between the nodes, providing explicit information regarding the properties of each node compared to other components [42], [43]. In the present study, we treated locations in the environment as nodes in a network, with the analysis aimed at unveiling the impact of each location (node) on the rats’ spatial behavior. Since the paths of travel in rats tend to converge at salient landmarks (e.g., objects [38]), we tested the rats in a round open field featuring 4, 8 or 12 objects, assuming that their paths would converge at these objects and thereby emphasize their potential as the network nodes. A rat can be either locomoting or not. During locomotion, rats do not perform large vertical or lateral movements, or activities such as grooming, with such movements or activities being performed during stops [44], [45]. This spatio-temporal separation allows us to describe locomotor behavior in terms of a sequence of stops at specific places [46]. In network analysis, this separation could facilitate the spatial location of the topologic analysis of each node (a cluster of stopping coordinates) and its connectivity with other nodes, and thereby represent spatial behavior as a network of locations. A prerequisite for the planned analysis was the definition of nodes (locations) based on the rats’ behavior. Considering the attraction of rats to objects in an open field [20], [37], [47], we analyzed the behavior of rats in symmetrical compared to asymmetrical object spacing. Previous studies had revealed that, for both humans and rats, wayfinding in symmetrical environments with a regular structure is easier than in environments with an irregular structure [28], [35], [48], [49], [50]. Accordingly, the rationale behind the comparison between symmetrical and asymmetrical environments was to discriminate between the topologic and spatial properties of the nodes. Specifically, we presumed that attraction to objects would be reflected in repeated visits or in the time spent at the objects, while symmetry and asymmetry were considered as spatial properties with potential impact on travel paths. Accordingly, in the symmetrical object layout, the topologic and spatial attributes coincided, whereas in the asymmetrical layout they diverged. Finally, it is interesting to note that network analysis seems to fit O’Keefe and Nadel’s [2], suggestion to view spatial representation as a set of connected places. Bearing this notion in mind, we sought behavioral means for the definition of locations in the spatial behavior of rats, and examined whether network analysis might reveal previously unknown facets of spatial behavior.

## Materials and Methods

### Animals

Male Wistar rats (n = 16; age 3 months; weight 250–300 g) were housed in a temperature-controlled room (21°C) with 12/12 h light/dark cycle (dark phase 8∶00 to 20∶00). Rats were held in standard rodent cages (40×25×20 cm; 2 rats per cage) with sawdust bedding, and were provided with free access to water and standard rodent chow. Each rat was marked with a waterproof marker on its tail, and acclimated to handling –10 minutes a day for one week. This study was carried out in strict accordance with the institutional guidelines for animal care and use in research. The study was approved by the Committee on the Ethics of Animal Experiments of Tel-Aviv University (permit L-10-013).

### Apparatus

**Figure 1:**
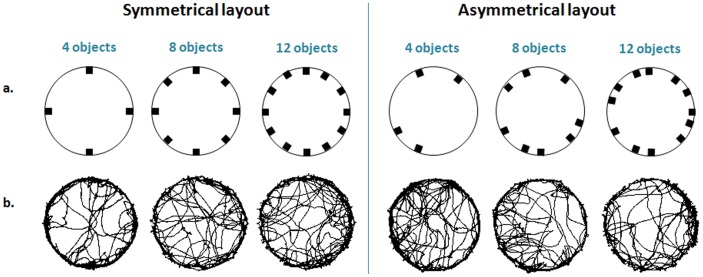
Object layout and paths of locomotion for an exemplary rat in each group. The location of 4, 8, and 12 objects in a symmetrical (left) and an asymmetrical (right) layout is depicted in the top row (a). The paths of locomotion for a single exemplary rat in depicted below for each layout (b).

Rats were tested in a round arena, 200 cm in diameter, surrounded by a 50 cm high tin wall. The arena was placed in a temperature-controlled (21±1°C) and light-proofed room. The arena floor was covered with a navy-blue PVC layer. During testing, the room was completely dark, illuminated only by infra-red light invisible to rats (Tracksys, IR LED Illuminator; UK, with a 830 nm wavelength filter). Trials were recorded by a video camera (Ikegami B/W ICD-47E, Japan) placed 2.5 m above the center of the arena, providing a top view of the entire arena. Footage was saved on a DVD device (Sony RDR-HXD 870, Japan). Each rat underwent three trials, with 4, 8, and 12 objects respectively, in only one of the following two object layouts (see Figure 1). Objects (black cement blocks; 6.5×6×6 cm), were placed in either a symmetrical or an asymmetrical layout along the arena walls. In the asymmetrical layout, distance between objects was established randomly, with at least 25 cm between objects, preventing the rats from touching two objects at the same time. Overall distance between objects was equal on average for both layouts in each trial.

### Procedure

Sixteen rats were randomly assigned to two groups of eight rats, each undergoing three trials in an arena with either a symmetrical or an asymmetrical layout of objects. Each rat was individually tested on alternate days with an increasing number of objects, starting with 4, then 8, and finally 12 objects. We did not include the counterbalanced paradigm (12, 8 and then 4 objects) since our previous studies had indicated that this procedure does not affect the results [23], [51]. At the beginning of each trial, a rat was placed at a fixed start location next to the arena wall, and its behavior was recorded for 20 minutes. The arena was wiped with detergent between successive trials. All testing took place during the dark phase when rats are most active.

### Data Acquisition and Analysis

#### Path analysis

For analysis, the arena was divided into the following virtual areas:

1. *Perimeter* - a 15 cm wide strip along the arena wall.
2. *Center* - the remaining central area of the arena (excluding the perimeter area).
3. *Object area* - a 25×25 cm square, around each object. Since objects were placed along the perimeter, object areas were within the perimeter area.

The paths of movement of the rats in these areas were tracked from the video files using ‘Ethovision XT 7’ (Noldus Information Technologies, NL), a software that provided the coordinates of the center of mass of the rat five times per second. The following parameters were extracted for further analysis with ‘Microsoft Excel 2007’:

1. *Distance traveled* - the cumulative metric distance (m) traveled over 20 minutes.
2. *Duration* - the time spent (min) at each of the arena areas.
3. *Travel between center and perimeter* - incidence of crossing between center and perimeter areas.
4. *Duration at an object* - the time spent (min) in an object area.
5. *Visits to an object* - the number of entries into an object area.

#### Network analysis

**Figure 2:**
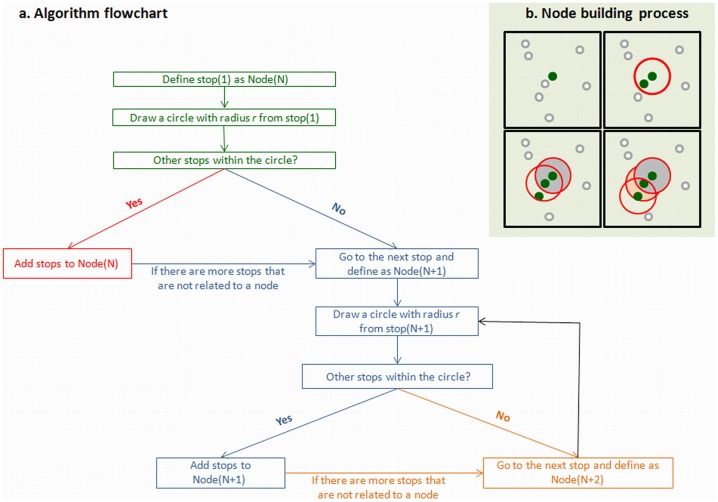
Building a node from stopping coordinates. The algorithm for transforming stopping coordinates into a network node (a) and a visualized process of building a single node (b).

**Figure 3:**
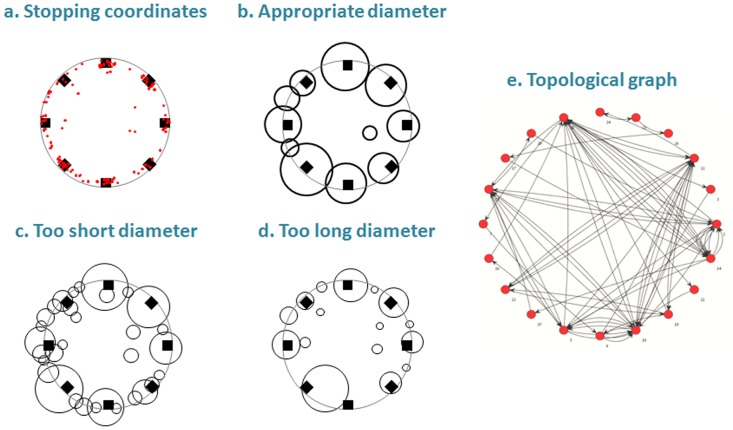
Building a network of places. The rationale for establishing the criterion of 12 cm diameter and the transformation of stopping coordinates into a network is illustrated for one rat. a. *Stopping coordinates: -* these are as the x-y coordinates of a single rat, as extracted from the tracking system (Ethovision). The large black circle represents the arena perimeter, each red dot represents a stopping coordinate at which the rat stopped for one second or longer, and the black squares represent the location of the objects. b. *Nodes under the application of a 12-cm circle around the additional stopping coordinate:-* As shown, with this diameter the nodes (circles) coincide with the objects and behavior. c. *Nodes under the application of a 9-cm circle around the additional stopping coordinate:-* As shown, with this diameter stopping coordinates around the same object split into several nodes, resulting in a mismatch between behavior and nodes. d. *Nodes under the application of a 14-cm circle around the additional stopping coordinate:-* As shown, with this diameter the bottom node encompasses the stopping coordinates of two objects (see the red dots of these objects in a.). e. *Topologic graph:-* The presentation of the network after the transformation of stopping coordinates into nodes (red circles). Arrows between nodes represent the links (passes) between nodes. Note that the location of a red circle does not represent the physical location of that node. Likewise, the circles that represent the nodes in b-d do not represent the real size of the node but the number of stopping coordinates included in that node.

For network analysis, behavior was considered as a set of locations and the transitions between these locations. In this representation, we defined local parameters that referred to the behavior within a specific location, and global parameters that referred to the behavior in the entire arena. Custom-designed software (‘Huldot’ by Michael Lieberthal) was used to identify locations of interest for the rats, as reflected by their X-Y stopping coordinates inside the arena. We defined a stop as no progression for at least 1 second. ‘Huldot’ is an algorithm based on the stopping behavior [44], [46], and its mathematical principles resemble the City Clustering Algorithm (CCA) [52], [53]. Although both ‘CCA’ and ‘Huldot’ deal with clustering spatial activities, the latter represents a novel approach to the study of spatial behavior in rats. Using the ‘Huldot’ algorithm, we identified the rat’s first stopping coordinate and defined it as node1. We then added to node1 all the stopping coordinates that were located at *d*≤*l* from the first stop (where *d* represents the measured distance between the stops and *l* represents a “unifying criterion” that was set to 12 cm – about half a rat’s body-length). We continued adding new stops to node1 until there were no more stops at *d*≤*l* from any of the stops included in node1. We then identified the rat’s next stop (not included in node1), defined it as node2, and repeated the process (Figure 2). The distance between stopping coordinates was calculated, and stopping coordinates within a 12-cm diameter were assigned to the same node. This diameter was found to be the best fit according to the following considerations: physically, this diameter (12 cm) had to be less than 14.5 cm (which is half the shortest distance between objects), as otherwise stopping coordinates at two adjacent objects could be attributed to one node at an intermediate distance between the two objects (Figure 3). The 12-cm diameter was also greater than 9 cm in order to prevent the splitting of stopping coordinates at the same object into two separate nodes (Figure 3). Within this diameter range, the 12-cm diameter was the best fit for all animals. Altogether, the algorithm provides a method by which to define locations in the spatial behavior of rats, offering a useful definition irrespective of network analysis. In the present study, however, this definition of locations (nodes) was a prerequisite for network analysis, enabling us to view the behavior as a network comprised of nodes (node = clusters of stopping locations), and of links between these nodes (link = pass from one node to another). Moreover, once the nodes and the links between them were established, it was possible to refer only to the topology of the behavior while ignoring the metric distance between actual stopping coordinates, and to analyze the behavior only in terms of the nodes and links that constituted the network (Figure 3b).

*Local network parameters: -* Once nodes and links had been defined, the following parameters were provided by ‘Huldot’ for each node in the network:

1. *Degree/Connectivity* (**k**) - the number of links that a node has with other nodes (i.e. the number of neighbors that a node has).
2. *Clustering coefficient* (**C**) - the number of actual links between the neighbors of a specific node, divided by the total number of possible links that could occur between them (“how many of a node’s neighbors are also each other’s neighbors”). The clustering coefficient (**C**) is a value between 0 and 1, representing the level of connections between all of a node’s neighbors (what portion of a node’s neighbors are themselves neighbors). This was calculated as follows: 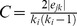 where 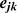 is the number of links between node ***i*** to other neighbors, and ***k_i_*** is the number of node ***i*** neighbors.
3. *Shortest path length* (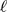) - the minimal number of nodes needed to be traveled in order to reach all the nodes in the network from a specific node.

The above parameters shed light on three different aspects of the nodes, indicating the relative importance of the nodes within the network. For example, a node with a high degree (**k**) and a low shortest path length (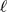) would be typical to a key node (hub) for travel within the network.

*Global network parameters:* While the above parameters refer to specific nodes, additional parameters were calculated for the entire network of each rat, as follows:

1. Total number of stops
2. Total number of nodes
3. Average network degree (**<k>**) - the average number of links per node (the average of the above degree values of all nodes). This parameter represents the connectivity of the network.
4. Average network clustering coefficient (**<C>**) - the average of the above local clustering coefficients of all nodes.
5. Average network shortest path length (**<l>**) - the average of the above shortest path lengths of all nodes. This parameter reflects the minimal number of nodes that needed to be traveled in order to reach from any node to any other node in the network.
6. Network density (**d**) - the ratio of the number of actual links divided by the number of theoretically possible links between all nodes.
7. “Key nodes” - nodes that encompass 10 or more stopping coordinates were defined as key nodes. The value of 10 stopping coordinates was set as a threshold based on ranking all nodes according to the number of stopping coordinates clustered within them. A two-fold difference from other nodes was noted in the layout with highest ranking nodes, which were thus separated and defined as key nodes, with 10 as their minimal number of stopping coordinates.

### Statistics

Data were compared by either a two-way ANOVA with repeated measures followed by a Tukey post-hoc test, or by means of a Student’s *t*-test. Alpha level was set to 0.05.

## Results

### Overall Activity in the Arena

Figure 1b presents the paths of progression of two exemplary rats, one for each of the two layouts in each of the three trials. As shown, in both object layouts and in all trials, activity was higher at the perimeter, with paths converging upon the objects. In the 4-object layout, rats in the asymmetrical layout crossed the arena center more frequently compared to the symmetrical layout. When objects were added to the perimeter, activity at the center of the symmetrical layout increased while in the asymmetrical layout activity at the center decreased. Indeed, as shown in Figure 1b, in the 12-object layout rats in the symmetrical layout crossed the arena center more frequently than rats in the respective asymmetrical layout. Overall, paths of progression differed between the layouts mainly at the arena center both due to symmetry layout (most apparent in the 4-object layout), and to increase in objects along the perimeter.

To test the effect of object layout and number on the level of activity, we performed a two-way ANOVA analysis of the total distance traveled in the arena. There was no significant difference between groups (F_1,14_ = 0.17; p = 0.688), but there was a significant difference between trials (F_2,28_ = 3.43; p = 0.047), and a significant interaction between groups and trials (F_2,28_ = 5.60; p = 0.009). The traveled distance did not differ between the two layouts, but did differ between trials (Table S1 of the supporting information). A Tukey HSD test revealed that, in the asymmetrical layout, rats traveled a shorter distance in the arena with 12 objects compared to that with 4 objects.

### Activity at the Arena Perimeter and Center

Since objects were placed only along the perimeter of the arena, we divided it into a perimeter area, comprising the layout of objects, and a center area, comprising the bare central sector (see ‘Methods’). The details of all activity parameters are provided in Table S1 of the supporting information. A two-way ANOVA analysis of the distance traveled at the perimeter revealed no significant difference between layouts (F_1,14_ = 0.13; p = 0.723), whereas there was a significant difference between trials (F_2,28_ = 4.66; p = 0.018). There was no significant interaction between groups and trials (F_2,28_ = 0.50; p = 0.610). A Tukey HSD test revealed that this difference arose from a shorter traveled distance at the arena perimeter with 12 objects compared to that with 4 objects. Altogether, rats in both layouts traveled a greater distance at the perimeter with 4 objects compared to 12 objects. As also shown in Table S1, a two-way ANOVA analysis of the distance traveled at the center revealed no significant difference between the two layouts (F_1,14_ = 0.09; p = 0.771), or between trials (F_2,28_ = 1.03; p = 0.371). However, the interaction between groups and trials was significant (F_2,28_ = 36.01; p<0.000). A Tukey HSD test revealed that when objects were added to the layout at the perimeter, distance traveled at the center increased in the symmetrical layout and decreased in the asymmetrical layout.

The difference in activity at the arena center was also manifested in the time spent there. A two-way ANOVA revealed that the time spent at the center did not differ between layouts (F_1,14_ = 0.38; p = 0.547), but did differ between trials (F_2,28_ = 9.01; p = 0.001). The interaction between groups and trials was not significant (F_2,28_ = 1.09; p = 0.349), but a Tukey HSD test revealed a difference between the symmetrical layout of 4 objects and that of 12 objects. This difference arose from the longer duration spent at the arena center in the symmetrical layout with 4 objects compared to that with 12 objects. Overall, as evident from the paths of progression (Figure 1b), the two groups differed mainly in the 4 object layout, with a higher activity level shown for the asymmetrical layout.

### Activity at the Objects

Table S1 presents data on the activity of the rats at the objects. As shown, in arenas with either symmetrical or asymmetrical object layout, with the increase in object number over the trials, the average duration of time spent at an object decreased, whereas the total duration spent at all objects increased. This was also replicated in the number of visits, which decreased per object but increased overall over trials. The Tukey HSD test revealed that the average time spent at an object and number of visits to objects decreased when objects were added, with significant difference between trials. In contrast, when pulled for all objects, the time spent and number of visits significantly increased between trials. In summary, rats tested in the symmetrical layout displayed an increase in distance traveled, duration, and number of visits at the arena center as objects were added to the perimeter; while rats tested in the asymmetrical layout displayed a decrease in traveled distance and no significant change in duration and number of visits at the center. In both layouts, rats traveled a greater distance at the perimeter with 4 objects compared to 12 objects, while the addition of objects decreased the duration and number of visits per object.

### Topologic Properties of Locomotor Activity

The impact of the environment became more obvious when the behavioral data underwent network analysis. In the first step, we constructed a topologic graph of the relations between locations in the environment, defined in ‘Methods’ as ***nodes*** (clusters of stopping coordinates within a 12 cm diameter), and the ***links*** (transitions) between these nodes (Figure 3). The detailed parameters of the network analysis are shown in Table S2 of the supporting information. There was no significant difference in the total number of stopping coordinates that represents the level of activity, but there was a significant difference in the spatial distribution of activity, as reflected in several significant differences in network parameters. First, the number of nodes was significantly affected by trials but not by object layout, and there was no interaction of layout x trial. A Tukey test revealed that the number of nodes in both layouts with 12 objects was higher than that with 8 objects. Implicit in this difference is that the spatial distribution of stopping coordinates among the nodes differed among trials when the number of objects was increased.

Second, the degree (connectivity) of the locations (nodes) did not differ between the layouts but did differ between trials. In the symmetrical layout the degree was higher in the layout with 8 objects compared to that with 4 objects, whereas in the asymmetrical layout the degree was higher with both 4 and 8 objects compared to that with 12 objects. Altogether, in terms of network properties, the number of nodes was higher in arenas with 12 objects. The degree (connectivity) of the network in the symmetrical layout was also affected by trials (increased number of objects) but not by group (symmetrical vs. asymmetrical object layout). The interaction of object-number X object-layout was nevertheless significant. A Tukey test revealed that in the symmetrical object layout, the degree with 8 objects was higher than with 4 objects, whereas in the asymmetrical object layout it was higher with 4 or 8 objects than with 12 objects. The clustering coefficient, which characterizes the overall tendency of nodes to aggregate, did not differ significantly between layouts and trials, ranging between 0.4 to 0.5 (Table S2). This value range indicates that the nodes indeed tend to aggregate in all trials and object layouts.

Third, another network parameter, the shortest path, reflects the topologic distance between nodes; that is, the minimal number of nodes that on average separate any node from another node in the network. This parameter did not differ significantly between the two layouts (Table S2 of the supporting information). However, in the asymmetrical layout the average of shortest path length increased in the 12-object layout compared to 4- and 8-object layouts. Finally, the network density (i.e. the ratio of existing links in the network to the number of possible links between nodes) did not differ significantly between layouts but did differ between trials. Specifically, the network density was lower in the asymmetrical layout with 12 objects compared to that with 8 objects. Overall, the main difference in the global topologic properties between the groups was manifested in the asymmetrical layout of 12 objects, which had the lowest connectivity and density and the highest path length between nodes.

### Key Nodes and Objects Location

Nodes that combined more than 10 stopping coordinates were considered as key nodes, with greater impact on the rats’ behavior. These key nodes comprised only 21%±2% of the total nodes, yet they accounted for 72%±3% of the total stopping coordinates. A two-way ANOVA analysis revealed no significant difference in the number of key nodes between groups, but a significant difference between trials. The interaction of object number X object layout was also significant. A Tukey HSD test revealed that rats tested in the symmetrical layout of 8 objects displayed more key nodes than in the symmetrical layout of 4 or 12 objects, and in the asymmetrical layouts of 8 or 12 objects.

**Figure 4:**
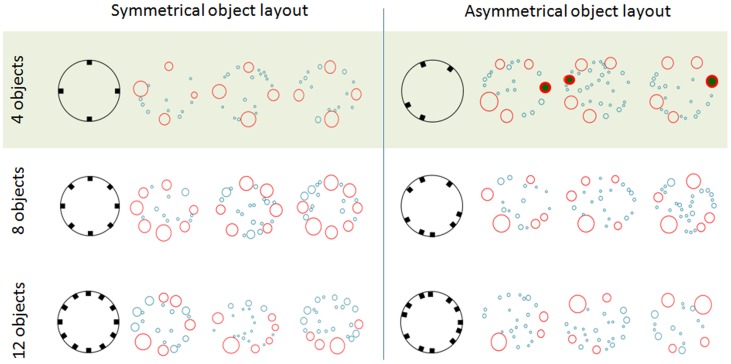
Physical location of the network nodes. For both the symmetrical (left) and asymmetrical arenas (right), the object layout is depicted in the left-hand column. The network nodes were placed in their respective physical location in the arena, and are shown for 3 rats in each object layout and object number. For each rat, the open circles represent the nodes in their physical location in the arena, and the diameter of the circle represents the number of stopping coordinates within each node (and not the physical area of the node). Key nodes are depicted in open red circles, whereas key nodes that are not located on objects are depicted in red circles filled with green. The rest of the nodes are depicted in light blue. As shown, in the asymmetrical layout with 4 objects, rats established a fifth node that is not located on an object.

When the physical location of the key nodes was scrutinized, all key nodes in the symmetrical layouts were located in close proximity to objects placed in the arena, regardless of object number (Figure 4). However, this gradually changed with the increase in number of objects in the asymmetrical layouts. In the asymmetrical layouts with 8 or 12 objects, all key nodes were located in close proximity to objects (Figure 4), as in the symmetrical layout. Finally, in the asymmetrical layout with 4 objects, all the rats had at least one key node away from the objects (Figure 4). Consequently, we set out to determine the physical properties of these key locations, and their impact in shaping spatial behavior. We thus focused on the rats tested with 4 objects in the asymmetrical layout, in which all rats had displayed key locations both on objects and away from objects, compared with the rats that were tested with 4 objects in the symmetrical layout, in which all key nodes coincided with objects.

**Figure 5:**
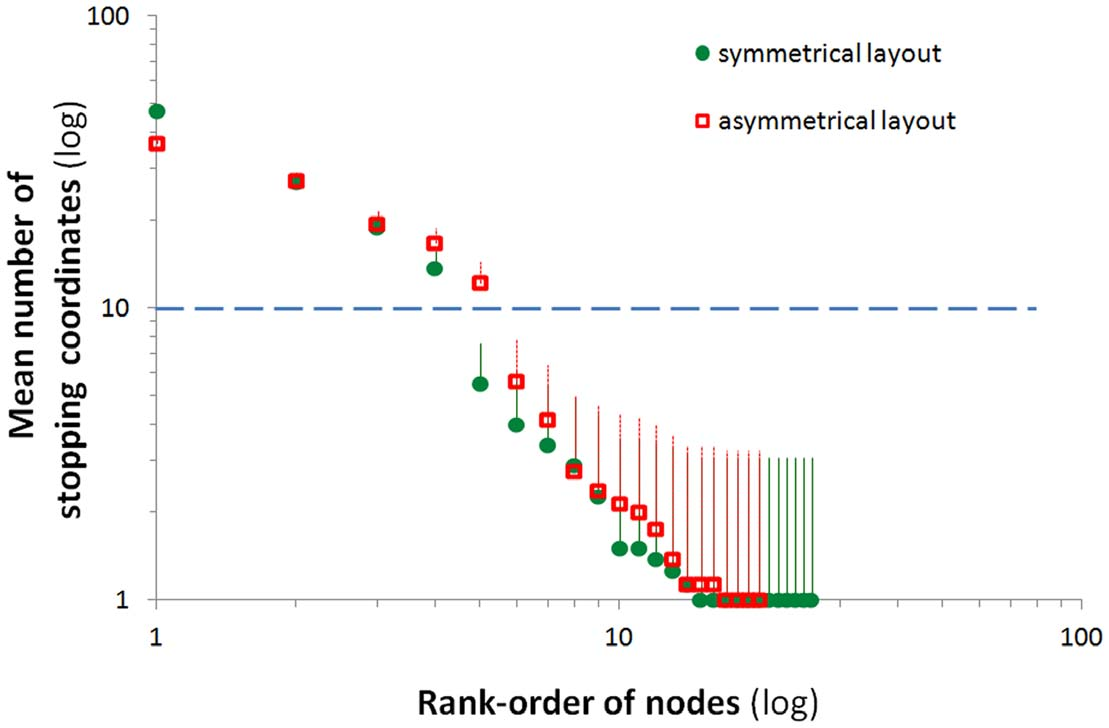
The distinction between key-nodes and other nodes. The nodes for each rat in the 4-object layout were ranked from high to low according to the number of stopping coordinates. The rank is depicted on the x-axis, whereas the mean (±SEM) number of stopping coordinates in each rank is depicted on the y-axis. Scale for both axes is logarithmic. The nodes above the dashed horizontal line are those that were considered as key nodes. As shown, there were four key nodes in the symmetrical layout compared with five key nodes in the asymmetrical object layout.

To further demonstrate the difference between the key nodes in the two layouts with 4 objects, we first ranked the nodes from highest to lowest according to the number of stopping coordinates clustered into each node. As shown in Figure 5, the four top ranks in the symmetrical layout and the five top ranks in the asymmetrical layout were distinctly different from all other nodes. This further reconfirmed our definition of ‘key nodes’ (see ‘Methods’), which in arenas with 4 objects included the first four top-ranked nodes in symmetrical object layout and the first five top-ranked nodes in asymmetrical object layout. The distinctive properties of the fifth-ranked node in the asymmetrical object layout were also obvious when compared with the fifth-ranked node in the symmetrical layout. Indeed, rats in the asymmetrical layout stopped more frequently at the fifth ranked node (t_14_ = 3.32; p = 0.005), and that node had a higher degree (t_14_ = 3.57; p = 0.003) compared to the symmetrical layout.

In the symmetrical layout with 4 objects, all four key nodes coincide with the objects (mentioned above). In the asymmetrical 4 object layout, there must therefore have been at least one “extra” node (out of the 5 key nodes) that could not coincide with an object. We found that all “extra key nodes” converged on either one of two specific physical locations in the arena perimeter. Both these locations had a common feature: their distance to the nearest physical object was similar to the distance between two adjacent physical objects (Figure 4). This location with no “physical object” thus apparently contained a “virtual object”. The location of this “virtual object” was between distant physical objects, at a distance adopted from the spacing of the real objects, so that the rats had divided the large gap between the objects through the aggregation of stopping coordinates at a “virtual object”. It should be noted that the rats that were tested in the asymmetrical layout had not been exposed beforehand to a symmetrical layout. Therefore, their tendency to add a “virtual” object reflects an inner property rather than the transmission of previous spatial knowledge. In other words, without the clear geometrical structure of a symmetrical layout, rats *de facto* created their own logical symmetrical order by adding “virtual” object(s).

## Discussion

Rats were tested in a round arena with either a symmetrical or an asymmetrical layout of 4, 8, or 12 objects located along the perimeter. Only subtle differences were found between rats tested in the two layouts in terms of spatial activity (travel distance, number of visits, etc). Nevertheless, network analysis revealed substantial differences in the rats’ behavior between the layouts. Specifically, by viewing the different locales at which the rats stopped as the network nodes, we found that in the arena with 4 objects the rats in both layouts established four key locations that coincided with the physical location of the objects, whereas in the asymmetrical layout rats established an extra key-location (and, rarely, two extra key-locations). The additional locations were spaced at a distance that was identical to the distance between other objects, as if the rats had introduced a ‘virtual object’ and thereby behaviorally imposed symmetry upon their spatial behavior in an asymmetrical environment. In the following discussion we first relate to our novel definition of a location in terms of spatial behavior rather than in terms of the mere physical attributes of the environment. We then discuss the various ways by which rats could simplify wayfinding, with emphasis on spatial symmetry. Finally, we highlight the potential of network analysis as a means for studying spatial behavior.

When a rat is introduced into an unfamiliar arena it soon establishes a ‘home base’, at which it stays for extended periods and to which it pays more visits compared to other places in the arena. From this home base the rat sets out for roundtrips in the arena, and its entire spatial behavior may be viewed as a sequence of consecutive roundtrips to the home base [44]. In the same vein, the network analysis that was applied in the present study revealed that one node (cluster of adjacent stopping coordinates) stood above all other nodes in the network in terms of the number of stopping coordinates (visits) and the number of other nodes with a direct link to that node (degree). This polarity of the network was more obvious in the asymmetrical layout, and was augmented with the increase in the number of objects. Indeed, in terms of the number of stopping coordinates, the first-ranked node comprised almost double the stopping coordinates than those of the second-ranked node in the symmetrical layouts (ratio of 1.76±0.35, 1.61±0.31, and 1.94±0.17 between first and second node for arenas with 4, 8, or 12 objects, respectively). In contrast to the steady ratio in the symmetrical layouts, the ratio between the first and second node in the asymmetrical layouts increased with the increase in number of objects (1.3±0.13, 2.71±0.42, and 3.19±0.85 for arenas with 4, 8, or 12 objects, respectively). Thus, the polarity among clusters of stopping coordinates was greater in the asymmetrical object layout and increased with the number of objects. In trials with 8 and 12 objects, distances between objects were shorter and the dominant (first-ranked) node often expanded over several objects. In other words, when objects were located in close proximity, rats often related to them as a unified location rather than several discrete locations. Implicit in the expansion of a location over several objects is that the behavioral span of a location does not necessarily coincide with the physical attributes of the environment. In this context, it should also be noted that the study of exploration in rats and other rodents has not yet found a means by which to define a location in spatial behavior. Rather, locations are defined by means of either an arbitrary grid that is imposed on the environment, dividing it into sectors [26], [44]; or alternatively, locations are marked by specific physical properties (wall, corner, shelter, salient landmark, object, etc; [20], [21], [35], [38]). The novelty in the present analysis thus lies in providing a means by which to define a location and its importance compared to other locations, as manifested in spatial behavior.

The question arises as to why polarization of activity was greater in the arena with the asymmetrical compared with the symmetrical object layout. To explain this aspect of spatial behavior we have borrowed the term ‘legibility’ [54], which was coined in urban planning to describe the ease with which parts of the environment may be recognized and organized into a coherent pattern [54]. In the context of the present study, the increase in the number of objects in the symmetrical layout intensified the legibility since more objects adhered to the same spatial regularity; and the more objects, the more regular and thus legible was the environment. Indeed, the increased legibility with the increase in the number of objects in the symmetrical layout was reflected in the greater activity in the arena center, as measured by the traveled distance, duration, and number of visits. In contrast, adding objects in the asymmetrical layout intensified the irregularity of the environment, resulting in a reduced legibility. This impact of legibility reinforces the findings from our previous studies, in which the activity of rats tested in a grid layout of objects was equally distributed over the entire arena; whereas the activity of rats that were tested in an irregular layout of objects displayed a polarized activity that was anchored at the location in which they had been introduced into the arena [28], [35].

The difference in legibility between the arenas with symmetrical and asymmetrical layouts may also account for the addition of a key node (“virtual object”) in the arena with an asymmetrical layout of 4 objects. By adding a “virtual object” and spacing it according to the average distance between other objects in the asymmetrical 4-object layout, the rats enforced a behavioral symmetry over the asymmetrical layout, virtually increasing the lower legibility of that layout. However, a prerequisite for adding a virtual object in an asymmetrical layout is that of object spacing that is large enough to accommodate the extra virtual object. This condition was met only in the 4-object layout, whereas in the asymmetrical layouts with 8 or 12 objects, objects were too close to each other and could not accommodate another “virtual object”. In that case, the rats did not rely on symmetry but, rather, polarized the environment, as reflected in a single location that dominated all others, with a cluster of numerous stopping coordinates (Figure 4). This effect was not replicated in the symmetrical layouts, where objects were equispaced and spatial relations were preserved despite the increase in object number, thus maintaining a high legibility.

As previously stated, it appears that the rats in the asymmetrical layout with 4 objects were attempting to achieve some sense of symmetry in the structure of the environment, raising the question of why symmetry is so important for spatial behavior. In the absence of distinctive landmarks, perfect symmetry may cause some wayfinding problems [55]. Nevertheless, in assessing building-layout complexity, it was found that symmetric elements are judged as simple and easily navigable [49]. It was also argued that the misalignment of cognitive frames of reference leads to way-finding problems and impairs the integration of spatial knowledge [50]. Indeed, it was argued that in regular environments with a rectangular street grid (e.g., Manhattan), navigation may rapidly lead to accurate survey knowledge. In contrast, in irregular environments such as Boston streets along the Charles river, accurate survey knowledge develops more slowly, being based solely on navigation [48]. This finding was followed up in rats, demonstrating that in an arena with an equispaced grid of objects rats tended to travel over a greater area and to visit more objects, compared to rats that were tested in the same arena but with an irregular layout of the same objects [35]. Thus, in both humans and rats, a regular structure of the environment is more legible than an irregular structure [28], [48]. Altogether, the symmetrical arena was more legible due to its regular layout, which provided the rats with a predictable heading and distance to the locations of the objects at which their stopping-coordinates clustered. In the lack of such physical structural symmetry, the rats acted upon the environment in order to make it more legible, by behaviorally establishing symmetry. It should be noted that by suggesting the behavioral establishment of symmetry, we do not claim that the rats equally travel in both halves of the arena, but that by repeatedly visiting a symmetrical location with no object they seem to be reconstructing a symmetrical image of the environment with the asymmetrical object layout, and this process seems to facilitate wayfinding.

It could be argued that the establishment of an extra key node was primarily affected by object spacing rather than by a quest for symmetry. This is not likely for two reasons. First, the location of the extra key node (“virtual object”) was at a distance shorter than that between objects in the symmetrical arena. Therefore, if object spacing was the main factor, rats (or at least some of them) would also establish extra key nodes in the symmetrical arena. Moreover, all the rats in the asymmetrical arena established the extra key location at a specific distance that formed symmetry, and not in other arbitrary locations, such as, for example, midway between two real objects. Further manipulations on object-spacing would probably discriminate between the impact of spacing and symmetry. Nevertheless, the present finding on the consistent establishing of an extra key node at a symmetrical location supports the view that it is symmetry and not spacing that primarily determines the location of the “virtual object”.

As noted above, the increase in the number of objects in a symmetrical layout was accompanied by increased activity in the center of the arena (greater traveled distance, longer duration, and more stops in the arena center). Conversely, activity in the center of arenas with an asymmetrical object layout decreased with the increase in number of objects. Activity in the arena center is considered as a measure of anxiety: the higher this activity, the lower the anxiety [56], [57]. This might be explained by the notion that an asymmetrical environment is less legible, and therefore the likelihood of becoming disoriented is greater. Indeed, disorientation involves a sense of anxiety [54], [58]. Accordingly, the greater center activity displayed by the rats in arenas with a symmetrical object layout may indicate that the increase in number of objects facilitated orientation and increased legibility. Consequently, there was a decrease in the rats’ anxiety which was manifested in the greater distance traveled at the center.

In addition to the notion of environmental legibility [54], regularity and symmetry of object layout (in terms of inter-object positioning and distance) also provide a strong configural organization that facilitates the construction of a stable and coherent representation [59], [60], [61], [62]. The symmetry and/or the geometry of the environment have a fundamental role in the establishment of spatial representation [63], [64]. It was suggested that humans and other animals first perceive the geometry of the environment, and then paste landmarks and other types of information into this geometric framework [63] (see however [65], [66], [67]), and unambiguous geometry facilitates spatial orientation. Indeed, it was shown that rats tested with equispaced objects in a grid layout could cover the entire area while progressing from object to object [35]. However, when patterns of geometry or symmetry are not easily recognized, such as in an arena with irregular object layout, rats need to restrict their travel in the environment to a limited sector, unless they can rely on other recognizable cues or patterns [28]. The present findings demonstrate that rats that were tested in an arena with 4 objects in an asymmetrical layout did not restrict their travel in the environment, due to of establishing behavioral spatial symmetry. For this, the rats established a location at a symmetric distance to other objects, and they visited that location at an incidence equivalent to that of locations with objects, as if referring to this location as a ‘virtual object’. The virtual objects thus bridge the gap between a desired legible order and the actual asymmetrical object layout. In other words, the rats established a behavioral symmetry in an asymmetrical environment, and thereby were able to travel throughout the asymmetrical arena as they did in the physically symmetrical arena. We suggest that this process was facilitated by means of odometry (or pedometry).

When traveling in a dark environment like that used in the present study, rats rely (solely or partially) on self-generated cues, until they acquire spatial representation of the environment [68], [69], [70], [71]. In order to gain a sense of the distance traveled, they may utilize odometry, which is a form of continuous integration of internal idiothetic cues (e.g. vestibular, kinesthetic). Estimating distance appears to depend on self-velocity [72]. For example, in bees, three-dimensional distance is measured by the optic-flow speed of spatial information to the eyes [73], [74]. In terrestrial locomotion in ants, odometry is primarily based on step counting, or ‘pedometry’ [75], [76]. Likewise, it was suggested that rats possess a sense for estimating distances [77], and since rats are mainly nocturnal, their odometry would probably be based on a sort of step counting (or, alternatively, on tactile flow). It should be noted that by testing the rats in a dark environment, they were forced to explore the environment sector-by-sector, whereas in the light rats can simply view the objects and approach them directly. (The situation of the rats in the dark arena thus simulated sighted humans in a large environment that they cannot capture visually but need to explore sector-by-sector). Altogether, estimating the distance between real objects in the present study could constitute the mechanism by which the rats established the location of the “virtual object”.

The novelty in the present study lies in the application of network analysis tools for the study of spatial behavior in free-moving rats. Using this approach, we were able to reveal interactions between locations in spatial behavior, and to distinguish between physical locations and behavioral locations. Notably, these interactions were not evident when behavior was analyzed by means of commonly used parameters such as traveled distance, number of stops, etc. Since network analysis is applicable to studies of exploration, or any process of building up an image of the environment, it could also be applied in the study of behavior employing other set-ups, such as a Barnes maze [78]. It cannot, however, be applied in the present form when behavior is not comprised of progressing and stopping, such as in the case of behavior studied in the Morris water maze [30]. Network-like topologic features in spatial behavior were previously noted by Poucet [79], who claimed that spatial representation is primarily built and processed as a topologic depiction of the environment. It was then shown that topologic features exist in spatial representation in rats [80]. Nevertheless, while previous studies have revealed that topologic features such as connectivity are present in the spatial behavior of rats, they did not utilize classical network parameters such as node degree, clustering coefficient, or shortest mean path, making it difficult to compare them to other network-related studies. The present study confirms Poucet and Hermman’s conclusions, and expands the study of behavioral topology by highlighting the network characteristics of exploratory behavior in free-moving rats with no physical constraints on their progression. Specifically, while Poucet and Hermman [80] used a maze in which the locations and their connectivity were pre-determined and fixed, rats in the present study were able to determine locations-of-interest and their importance, as reflected in the connectivity and the number of stopping coordinates at each location. As noted above, a prerequisite for network analysis was the definition of the network’s nodes, and this acted as an impetus for us to develop a novel perspective on the ongoing debate of what is a ‘location’ in behavior. The novelty in the present study lies in adding a behavioral dimension for a “location”, which is a term that has been mostly discussed according to its physical attributes, as well as its neurobiological correlates – the place cells, border cells, and grid neurons. In this sense, the present methodology enables us to define a location behaviorally and to assess its importance, even when such a location does not have any distinctive physical properties.

## Supporting Information

Table S1 **Activity parameters.** Mean (±SEM) of the parameters of activity (left-hand column) are depicted for symmetrical and asymmetrical object-layouts with 4, 8 and 12 objects. The results of an analysis of variance with repeated measures are depicted on the right of the table for each parameter, for the comparison of symmetrical and asymmetrical groups (‘group effect’), for the number of objects (‘trial effect’) and the interaction of groups and trials (‘interaction’).
(DOCX)

Table S2 **Network parameters.** Mean (±SEM) for the parameters of network analysis that are listed on the left-hand column, are depicted for symmetrical and asymmetrical object layouts of 4, 8, and 12 objects. The results of an analysis of variance with repeated measures are depicted on the right of the table for each parameter, for the comparison of symmetrical and asymmetrical groups (‘group effect’), for the number of objects (‘trial effect’) and the interaction of groups and trials (‘interaction’).
(DOCX)
