# Supplementary material for: Network Analysis of Rat Spatial Cognition: Behaviorally-Established Symmetry in a Physically Asymmetrical Environment
Source: PLoS One. 2012 Jul 18;7(7):e40760. doi: 10.1371/journal.pone.0040760 (PMC3399894; doi:10.1371/journal.pone.0040760)
Supplement: Table S1 — Activity parameters. Mean (±SEM) of the parameters of activity (left-hand column) are depicted for symmetrical and asymmetrical object-layouts with 4, 8 and 12 objects. The results of an analysis of variance with repeated measures are depicted on the right of the table for each parameter, for the comparison of symmetrical and asymmetrical groups (‘group effect’), for the number of objects (‘trial effect’) and the interaction of groups and trials (‘interaction’). (DOCX) [file pone.0040760.s001.docx]

**Table S1**:

|  | **Symmetrical layout of objects** | | |  | **Asymmetrical layout of objects** | | |  | Group effect  (F_1,14_; p) | Trial effect  (F_2,28_; p) | | Interaction  group x trial  (F_2,28_; p) | |  |
| --- | --- | --- | --- | --- | --- | --- | --- | --- | --- | --- | --- | --- | --- | --- |
|  | 4 objects | 8 objects | 12 objects |  | 4 objects | 8 objects | 12 objects |  |  |  |  |  |  |  |
| Distance traveled at the arena (m) | 105.5 ± 6.3 | 109.8 ± 4.1 | 108.4 ± 4.2 |  | 122.5 ± 9.1 | 110.9 ± 5.7 | 99.1 ± 7.6 |  | 0.2; 0.688 | | **3.4; 0.047** | | **5.6; 0.009** | |
| Distance traveled at the perimeter (m) | 85.5 ± 6.3 | 84.8 ± 4.6 | 77.9 ± 3.8 |  | 91.8 ± 7.4 | 86.1 ± 4.8 | 76.7 ± 5.3 |  | 0.2; 0.723 | | **4.7; 0.018** | | 0.5; 0.610 | |
| Distance traveled at the center (m) | 19.9 ± 2.6 | 24.9 ± 2.1 | 30.5 ± 2.5 |  | 30.7 ± 2.5 | 24.8 ± 2.0 | 22.4 ± 2.3 |  | 0.1; 0.771 | | 1.0; 0.371 | | **36.0; <0.000** | |
| Travel between center-perimeter (#) | 24.1 ± 2.7 | 30.5 ± 2.0 | 30.9 ± 2.0 |  | 32.5 ± 2.9 | 34.1 ± 2.0 | 33.5 ± 3.1 |  | **7.7; 0.015** | | 1.7; 0.195 | | 0.8; 0.464 | |
| Duration at the center (min) | 1.8 ± 0.4 | 2.2 ± 0.3 | 3.1 ± 0.3 |  | 1.9 ± 0.3 | 2.2 ± 0.3 | 2.5 ± 0.2 |  | 0.4; 0.547 | | **9.0; 0.001** | | 1.1; 0.349 | |
| Duration per object (min) | 1.4 ± 0.1 | 0.7 ± 0.0 | 0.5 ± 0.0 |  | 1.3 ± 0.1 | 0.6 ± 0.1 | 0.5 ± 0.1 |  | 0.1; 0.841 | | **217.6; <0.000** | | 0.1; 0.932 | |
| Duration at all objects (min) | 5.5 ± 0.3 | 5.9 ± 0.3 | 6.4 ± 0.5 |  | 5.5 ± 0.3 | 5.6 ± 0.5 | 6.2 ± 0.6 |  | 0.1; 0.790 | | **3.4; 0.047** | | 0.1; 0.909 | |
| Visits per object (#) | 20.7 ± 1.6 | 16.1 ± 0.8 | 12.3 ± 0.4 |  | 24.1 ± 1.6 | 16.8 ± 0.9 | 13.5 ± 0.4 |  | 2.7; 0.124 | | **70.1; <0.000** | | 1.5; 0.244 | |
| Visits at all objects (#) | 82.9 ± 6.4 | 128.5 ± 6.6 | 148.2 ± 5.2 |  | 96.2 ± 6.3 | 134.7 ± 7.1 | 161.5 ± 5.1 |  | 2.8; 0.118 | | **126.6; <0.000** | | 0.5; 0.622 | |
