# Supplementary material for: Network Analysis of Rat Spatial Cognition: Behaviorally-Established Symmetry in a Physically Asymmetrical Environment
Source: PLoS One. 2012 Jul 18;7(7):e40760. doi: 10.1371/journal.pone.0040760 (PMC3399894; doi:10.1371/journal.pone.0040760)
Supplement: Table S2 — Network parameters. Mean (±SEM) for the parameters of network analysis that are listed on the left-hand column, are depicted for symmetrical and asymmetrical object layouts of 4, 8, and 12 objects. The results of an analysis of variance with repeated measures are depicted on the right of the table for each parameter, for the comparison of symmetrical and asymmetrical groups (‘group effect’), for the number of objects (‘trial effect’) and the interaction of groups and trials (‘interaction’). (DOCX) [file pone.0040760.s002.docx]

**Table S2**:

|  | **Symmetrical layout of objects** | | |  | **Asymmetrical layout of objects** | | |  | Group effect  (F_1,14_; p) | Trial effect  (F_2,28_; p) | | Interaction  group x trial  (F_2,28_; p) | |  |
| --- | --- | --- | --- | --- | --- | --- | --- | --- | --- | --- | --- | --- | --- | --- |
|  | 4 objects | 8 objects | 12 objects |  | 4 objects | 8 objects | 12 objects |  |  |  |  |  |  |  |
| Total number of  stopping coordinates | 137.1 ± 6.6 | 157.5 ± 7.0 | 153.1 ± 5.5 |  | 143.5 ± 5.0 | 145.1 ± 6.8 | 135.6 ± 10.9 |  | 2.2; 0.157 | | 1.2; 0.327 | | 2.7; 0.086 | |
| Number of nodes | 19.6 ± 1.7 | 21.5 ± 1.7 | 24.5 ± 2.0 |  | 22.3 ± 1.5 | 18.6 ± 1.8 | 25.0 ± 1.2 |  | 0.0; 0.954 | | 5.0; **0.014** | | 1.5; 0.230 | |
| Average network degree | 6.9 ± 0.3 | 8.4 ± 0.5 | 7.2 ± 0.4 |  | 7.9 ± 0.5 | 7.5 ± 0.7 | 5.5 ± 0.4 |  | 1.1; 0.307 | | 10.9; <**0.000** | | 8.1; **0.002** | |
| Average network clustering coefficient | 0.5 ± 0.1 | 0.4 ± 0.1 | 0.4 ± 0.1 |  | 0.5 ± 0.0 | 0.5 ± 0.1 | 0.4 ± 0.1 |  | 0.3; 0.562 | | 2.4; 0.113 | | 1.7; 0.197 | |
| Average network shortest path length | 2.8 ± 0.2 | 2.9 ± 0.1 | 3.1 ± 0.2 |  | 2.8 ± 0.1 | 2.8 ± 0.2 | 3.4 ± 0.1 |  | 0.2; 0.643 | | 7.6; **0.002** | | 2.2; 0.134 | |
| Network density | 0.4 ± 0.0 | 0.4 ± 0.1 | 0.3 ± 0.0 |  | 0.4 ± 0.0 | 0.5 ± 0.1 | 0.3 ± 0.0 |  | 0.3; 0.596 | | 10.7; <**0.000** | | 0.8; 0.439 | |
| Number of key nodes (containing more than 10 stops) | 3.6 ± 0.2 | 6.0 ± 0.3 | 4.4 ± 0.5 |  | 4.9 ± 0.2 | 4.2 ± 0.4 | 3.5 ± 0.5 |  | 2.4; 0.143 | | 6.7; **0.004** | | 10.4; <**0.000** | |
